# Node Survival in Networks under Correlated Attacks

**DOI:** 10.1371/journal.pone.0125467

**Published:** 2015-05-01

**Authors:** Yan Hao, Dieter Armbruster, Marc-Thorsten Hütt

**Affiliations:** 1 Department of Mathematics and Computer Science, Hobart and William Smith Colleges, Geneva, New York, United States of America; 2 School of Mathematical and Statistical Sciences, Arizona State University, Tempe, Arizona, United States of America; 3 School of Engineering and Science, Jacobs University, Bremen, Germany; Wake Forest School of Medicine, UNITED STATES

## Abstract

We study the interplay between correlations, dynamics, and networks for repeated attacks on a socio-economic network. As a model system we consider an insurance scheme against disasters that randomly hit nodes, where a node in need receives support from its network neighbors. The model is motivated by gift giving among the Maasai called Osotua. Survival of nodes under different disaster scenarios (uncorrelated, spatially, temporally and spatio-temporally correlated) and for different network architectures are studied with agent-based numerical simulations. We find that the survival rate of a node depends dramatically on the type of correlation of the disasters: Spatially and spatio-temporally correlated disasters increase the survival rate; purely temporally correlated disasters decrease it. The type of correlation also leads to strong inequality among the surviving nodes. We introduce the concept of disaster masking to explain some of the results of our simulations. We also analyze the subsets of the networks that were activated to provide support after fifty years of random disasters. They show qualitative differences for the different disaster scenarios measured by path length, degree, clustering coefficient, and number of cycles.

## Introduction

Modern society is highly interconnected and depends in myriad ways on the existence and stability of its supporting networks. Be they infrastructure networks like power networks, telecommunication networks, water and transportation systems or social, financial, business and personal support networks—all of them are essential for a functioning society. At the same time all these networks exist in dynamic environments that are subject to smaller and larger disruptions created by natural forces (earthquakes, winter storms, draughts) or man made disasters as in wars, financial meltdowns or terrorist attacks.

In recent years the stability of such networks under cascading failure has been an important research topic. Dorogovtsev and Goltsev [1] discuss various different failure models in their 2008 review of critical phenomena in networks, focussing on sandpile and avalanche models in a variation of the Bak-Tang-Wiesenfeld [2] model. A different approach has been taken by Motter and Lai [3] who developed a basic scenario for a cascading failure: For a given network, limiting load thresholds are assigned to the nodes. Then a randomly selected node is attacked and deleted from the network and its load is distributed over neighboring nodes leading some of them to overload and collapse. In that way, a cascade of failures is generated that runs through the network. The failure is measured by the extend that the giant component in the network survives the attack. Motter and Lai discuss the susceptibility of different network topologies under random and targeted attacks. Other models for cascading failures try to stay closer to power systems networks and study different spreading mechanisms [4]. More recently, the interdependence of failures in different networks (e.g. a failure in the power system leading to a failure in the internet network) has been shown to be even more fragile than single networks by themselves [5].

In all these cases, the fundamental issue is the vulnerability of a heterogeneous self-organized network against single, often relatively small events. In contrast, the current paper aims to understand the impact of multiple attacks on a self-organized network. Specifically we are interested in the influence of spatially, temporally and spatio-temporally correlated attacks on a network. As a model system we consider the insurance scheme against disasters, based on networks offering mutual help in need, developed by the Maasai tribe in East Africa [6].

### Osotua

The Maasai are a pastoralist society that have developed a need based risk pooling system to deal with the impacts of natural (predominantly draught) or man made (warfare, cattle rustlers) disasters impacting their herds [7]. The system is called Osotua and involves a network of mutual relationships. An individual whose herd has been decimated below a sustainable level will ask his Osotua neighbors for help in the form of a gift of cattle to replenish his own herd up to a sustainable level. There is a social obligation to provide help, if possible, and help will only be requested up to the verifiable level of need. Aktipis et al. [6] developed an agent-based model formalizing the Osotua rules and simulated a society build upon pairs of Osotua partners. They simulated the impact of disasters which occur randomly in time upon randomly chosen individuals and their herd. They show that support based on Osotua principles leads to higher herd survival than simulations without transfer gifts or with probabilistic transfers.

Hao et al [8] extended the agent based simulation analysis to study the impact of the size and topology of Osotua networks on the herd survival rates of a group of participants in the Osotua scheme. They specifically studied the influence of the total number of participants in the network, of the mean degree of the network and of the asking-for-help-rules on the survival rate of a herd for randomly occurring disasters.

### Goals

The present paper studies the influence of repeated correlated disasters on a stylized dynamical model on a network. The specific dynamics that we are studying builds on the agent based simulations for an Osotua risk pooling system and keeps the Osotua interactions as coded in [6] and [8]. However, we are not interested in possible anthropological results but we are fundamentally interested in the impact of correlated disasters on a network based system. There may be some useful lessons to learn from our study on the resilience of Osotua systems in an environment characterized by global warming and the related increase in length and frequency of draught periods. Similarly, there are contact points to research related to catastrophic risk management and insurance risk theory. However, we do not know enough about the socio-economic situation of the Maasai to be able to make sound predictions or suggestions for their risk-pooling scheme, nor do we want to develop a full insurance risk theory for correlated disaster events. Our aim is to present a study of the interplay between correlations, dynamics and networks.

### Results

The influence of correlated disasters on the survival rates of herds depends dramatically on the type of correlation. Even without the Osotua network, increasing spatial correlation will monotonically (strongly) increase the survival rates whereas strong temporal correlations leads to a (smaller) decrease of survival. We consider four cases: A baseline case with weak spatial and weak temporal correlations, a case with strong temporal and weak spatial correlations, a case with strong spatial and weak temporal correlations and a case with strong temporal and strong spatial correlations. We refer to them as the no correlation case, the temporal correlation case, the spatial correlation case and the spatio-temporal correlation case, respectively.

Correlation also leads to strong inequality among the surviving herds: For disasters with strong temporal correlations and weak spatial correlations the herd size stays marginal whereas spatial correlations increases the herd sizes. Interestingly, the surviving herds for spatio-temporal correllations are as big as the ones for only spatial correlations. These results are presented and discussed in detail in Section 4.3.

Section 2 discusses the setup for the correlated disaster events, section 3 presents the details of the agent simulations and section 4 shows how the survival rates are modified by the network and especially by the inhomogeneity of the network. Section 4.3 presents heuristic explanations for some of the simulation results. Section 4.4 shows how the disaster activated support flows present a dual view of the social support networks that reflects the correlation of the disasters. Section 5 concludes with some general lessons learned and an outlook on further work.

## Correlations

Proverbially, disasters rarely strike once, they come in bursts with spatial or temporal correlation, or both. One important natural disaster of that type is a drought: A drought event has a spatial extend and a temporal duration. It has enduring effects on an area for a number of consecutive years. Consequently, if an individual’s livelihood is hit by a drought this year, it is highly likely that this person will suffer and need help for several years in a row. Additionally people living nearby in similar circumstances will also, with high probability, be affected by the draught. Hence the random events of being affected by droughts are spatially and temporally correlated.

Earthquakes are examples of recurring disasters that are predominantly spatially correlated. They occur randomly in time but are associated with certain locations determined by geologic fault lines. In the context of an Osotua network, an individual living in a marginal ecological domain will be the one always in trouble when an ecological disaster hits. Hence, a spatially heterogeneous support network should be a good insurance scheme against spatially correlated disasters.

The typical example for the other extreme case of a predominantly temporal correlation of disasters is related to a general economic downturn. A strong recession or depression of economic activities tends to affect all parts of a country more or less uniformly but has a lingering effect in time. If a large percentage of the population needed assistance this year, the probability that next year will also be a bad year is very high. Here spatial heterogeneity should have a marginal influence on the effectiveness of an insurance scheme.

**Fig 1:**
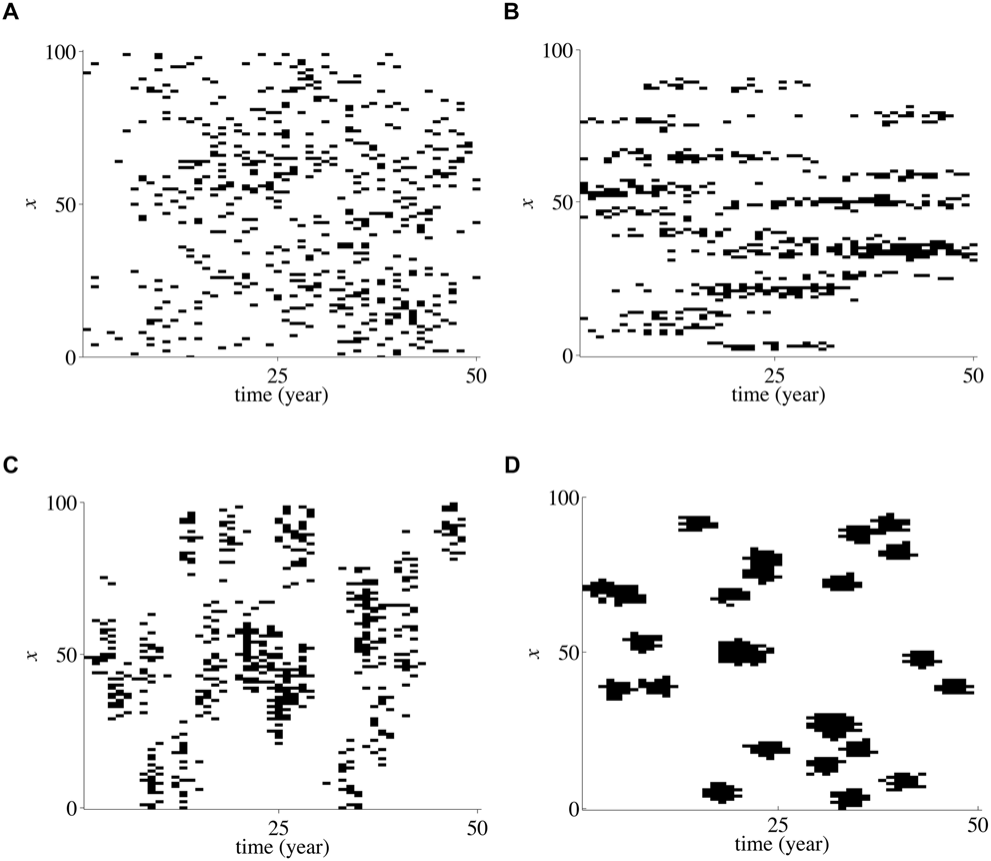
Samples of random events for four correlation scenarios. The horizontal axis represents time up to 50 years. The vertical axis represents a spatial grid. The total number of events is 500 for each panel. *σ*_*t*_ and *σ*_*x*_ are the variances in the spatial and temporal domain defining the level of correlation (see the detailed discussion in Section 2).

Fig 1 gives four visual samples of correlated disasters in a 2-dimensional space-time rectangle. Fig 1(a) shows disaster events randomly and independently distributed in space and time. Specifically the disasters are uniformly distributed in space and and follow a Poisson distribution in time. Fig 1(b), 1(c) and 1(d) demonstrate disaster events that are spatially correlated, temporally correlated and correlated both ways, respectively. Notice that the number of disasters in all the panels is the same but that the correlations lead to inhomogeneous visual patterns. When disasters are spatio-temporally correlated, they occur in clusters, or ellipses in Fig 1, spatially correlated disasters occur in horizontal line segments, temporally correlated disasters show up as vertical line segments.

Algorithmically, we employ two-dimensional Gaussian distributions to generate random correlated disaster events. Keeping the total number of disaster events, *N*, the same, we determine *M* event clusters and distribute them randomly in the same manner as the uncorrelated case in the two dimensional rectangle giving us the set of disaster events 𝓓 = {(*t*_1_, *x*_1_), (*t*_2_, *x*_2_), (*t*_*M*_, *x*_*M*_)}. Using (*t*_*i*_, *x*_*i*_) as the mode, the probability of a close-by point having a disaster is decided by a two-dimensional discretized Gaussian distribution. The probability landscape is consequently a M-modal distribution where each mode locally has the shape of a two-dimensional Gaussian. If the events in 𝓓 get too close, the Gaussians will overlap and increase the probability of further events in the overlap regions. Notice that the integral over the entire probability landscape is *M*, this probability landscape is consequently sampled without replacement for *N*/*M* times so that the expected total number of events is *N*.

The correlation lengths and the correlations strength between random events in(t,x)-space are therefore measured by the standard deviations of the Gaussian distributions *σ*_*t*_, *σ*_*x*_ visually representing the length of principal axes of the ellipses in Fig 1. As *σ* → 0 the Gaussians become *δ*-distributions of strength *N*/*M* representing *M* random clusters of *N*/*M* highly correlated events in space and time. As *σ* increases the correlation length increases but the correlation itself decreases. Correlation and correlation length depend on the number of clusters, the size of the simulation rectangle and the spatial and temporal discretization. Hence, since the variance of the Gaussian clusters is visually very intuitive and is very close to the algorithm that generates our disaster events, and since we are only interested in the qualitative response of the survival dynamics to changing correlations, we present the dependence of the survival rates for the simulated agents as a function of *σ*_*t*_, *σ*_*x*_ in the subsequent sections.

## The agent simulation

The Osotua agent simulations mostly follow the algorithm described in [8]. We recapitulate the main points here.

### The social network

We presume (without ethnological evidence) that Osotua support networks are Watts-Strogatz random networks [9]. Each random network is generated by randomly rewiring a fraction *β* of the edges of a homogeneous network while keeping the average degree of the network *k* = 4 unchanged. As *β* increases, the spatial heterogeneity of the network increases. We keep the size of the network at 100 nodes and typically choose two values of *β*: A spatially almost homogeneous network with *β* = 0.2 and a spatially strongly heterogeneous network with *β* = 0.8. In order to average out a specific network structure, we run all simulations with between 100 and 1000 randomly rewired copies for each experiment. Note that we use the rewiring probability *β* as a means of varying the amount of spatial heterogeneity, rather than generating a random network with small-world properties in the sense of [9]. The small world property is not relevant here, since there is not transport across the whole network.

### Correlated disaster events

By default we create on average *N* = 500 disasters in *M* = 40 clusters. The number of disasters in each cluster is a Gaussian random variable with a mean of $N_{event}=\frac{500}{40}=12.5$ distinct events and a variance of one. We will simulate a 50 year timespan in yearly increments on a one dimensional ring with 100 locations. We generate the time and location of disasters using the *bivariate normal* generator from Matlab by initializing a disaster matrix of size 100 × 50 with zeros. We then create *M* samples from a uniform distribution on the rectangle (1,100) × (1,50) representing the uncorrelated disaster centers. For each disaster center we create a bivariate normal distribution with standard deviation (*σ*_*t*_, *σ*_*x*_) and sample each distribution multiple times to obtain *N*_*event*_ distinct samples at the locations (*t*_*i*_, *x*_*i*_),1 ≤ *i* ≤ *N*_*event*_ and update the value of the disaster matrix at (*s*_*i*_, *t*_*i*_),1 ≤ *i* ≤ *N*_*event*_ to one.

### Osotua agents

Each node of the social network is occupied by an Osotua agent and his herd of cattle. Links in the network are bi-directional and represent a node’s Osotua neighborhood, i.e. the individuals that have mutual obligations to help each other. We are treating the index of a node in the homogeneous ring network, prior to rewiring, as a physical spatial variable. Hence disasters centered on a node *x*_*i*_ will affect the neighboring nodes *x*_*i*−1_, *x*_*i*−2_, *x*_*i*+1_, *x*_*i*+2_ etc, whether they are linked in an Osotua neighborhood with the node *x*_*i*_ or not. Highly spatially heterogeneous networks tend to have links that may reach outside the correlation length of a disaster center.

We formalize the Osotua gift giving schemes in the following ways:

1. The minimum sustainable cattle herd size is 64.
2. Asking rule: Individuals make a request for cattle once a year only if their current holdings are below the minimum herd size.
3. Giving rule: Individuals give what is asked, but not so much as to put the giver’s cattle holdings below the minimum herd size. An individual who cannot honor the full request without falling below the minimum herd size will give nothing. If asked, an individual may give multiple gifts.
4. Individuals make a request to a specific one of their Osotua partners chosen randomly with equal probability.

### Simulation

For each random social network the following simulation is repeated 1000 times by default: The simulation is initialized with an initial herd size for all agents of 70. Each year, each node experiences a random increase in their herd size with mean 3%. For a given disaster matrix, every year, the disasters are allocated to nodes. If year n is a disaster year for individual i, a random number $l_{i}^{\left(n\right)}\sim 𝓝(30\%,{\left(10\%\right)}^{2})$ is drawn to decide the percentage of the herd that is lost in this year. If, after eliminating the loss, the herd is below threshold, an Osotua request is made and if possible the herd is restored to the minimum sustainable size of 64 by a gift from an Osotua partner. A node will be eliminated, if its herd falls below the threshold for two consecutive years. The simulation is continued for 50 years and the node survival rate, the herd size per survivor node, and the location of the surviving nodes is registered. Averages over the 1000 simulations and the 100–1000 random networks are reported.

## Survival rates

### Survival rates for isolated nodes

**Fig 2:**
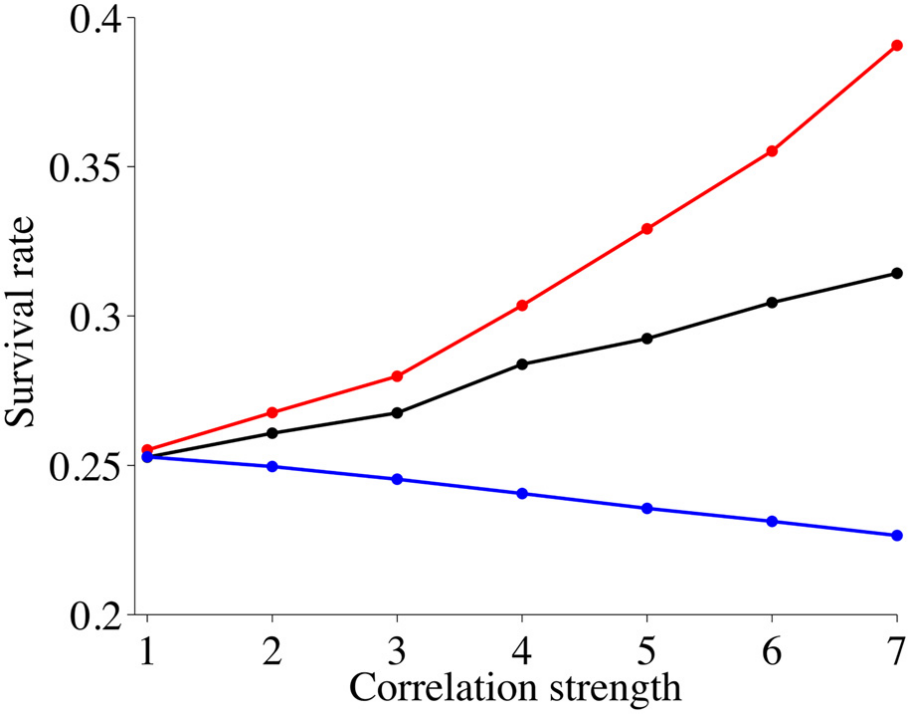
Average survival rates after 50 years for isolated nodes as a function of changing disaster variances and different disaster correlations. Red curves describe spatial correlations among disasters, black curves represent spatial-temporal correlations and blue curves represent temporal correlations. The correlation strength is defined in detail in the main text in Section 4.

As a benchmark we study a ring of nodes that are unconnected, i.e. without a gift giving scheme. Fig 2 shows the survival rates of herds at nodes after 50 years of disasters. The x-coordinate in Fig 2 is a measure of the standard deviation of the Gaussians centered at the *M*-modes of the probability distribution. Specifically *x* = *p* indicates *σ*_*x*_ = 2^−(*p*−1)^64 or *σ*_*t*_ = 2^−(*p*−1)^32, i.e. *x* = 1 describes a distribution with a standard deviation of 64 in space and 32 in time, i.e. the distribution approaches uniformity over the simulation rectangle of 100 spatial points and 50 years. Hence, correlation increases as the x-coordinate increases.

We see that correlations in these disasters have a huge effect on the survival rates: For strong spatial correlations the survival rate is almost twice as high as for strong temporal correlations, with spatio-temporal correlations staying between the two extremes.

### Survival rates for nodes in networks

**Fig 3:**
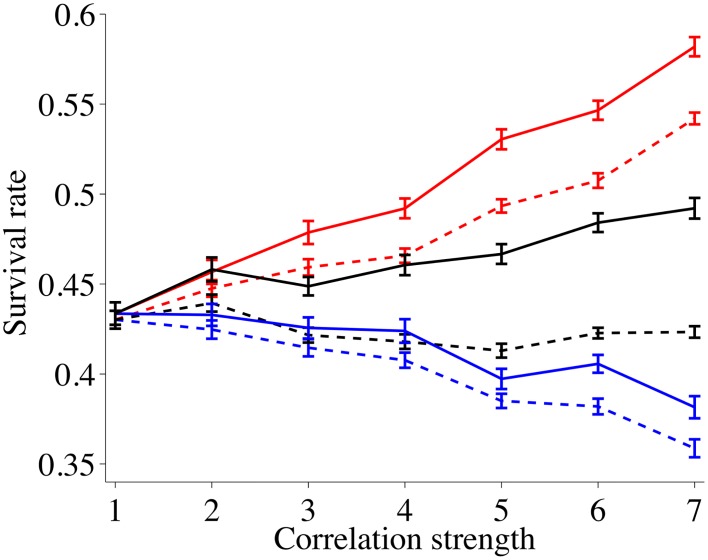
Average survival rates after 50 years for networked nodes as a function of the correlation strength among disasters. Solid lines describe networks that are very inhomogeneous (*β* = 0.8), dashed lines describe networks that are more homogeneous (*β* = 0.2). Red curves describe spatial correlations among disasters, black curves represent spatial-temporal correlations and blue curves represent temporal correlations.

Fig 3 shows that the network does not affect the relative impact of correlations on the survival rates compared to Fig 2: Increasing the spatial correlations leads to significantly higher survival, increasing temporal correlations leads to weakly lower survival and increasing both leads to a weak increase in survival. However, we can discern three major impacts related to the existence of a support network and their spatial heterogeneity: i) Support networks lead to more than 50% higher survival rates. ii) Spatially inhomogeneous networks lead to higher survival rates for all disaster correlations. iii) Spatially inhomogeneous networks show the highest gain in survival relative to homogeneous networks for spatio-temporal correlations.

### Disaster masking

In the case without a network, since nodes will not get any help from their neighbors, the differences in the survival rates are entirely due to the effect of the correlation of the disasters. In particular, the high survival rate for the spatially correlated disasters can be explained by an effect we call *masking*. Spatially correlated disaster events are highly likely to strike the same (small) group of nodes while the majority of the nodes keep growing their herds. Hence these disasters kill a small, unfortunate group but leaves the rest to grow. In addition, since disasters strike the same spatial locations, it his highly likely that disasters that happen late in the 50 year time period, hit locations where the herds were eliminated at an early stage of the simulation. Hence many programmed disasters are not effective since they hit the spots that have no live herds at all.

This *masking* effect is much smaller in temporal correlations: Disasters are widely distributed in space and hence are much less likely to hit an area where most of the herds have been eliminated by previous disasters. Since spatio-temporally correlated disasters have the same spatial extensions as the spatial disasters, masking is highly likely also.

**Fig 4:**
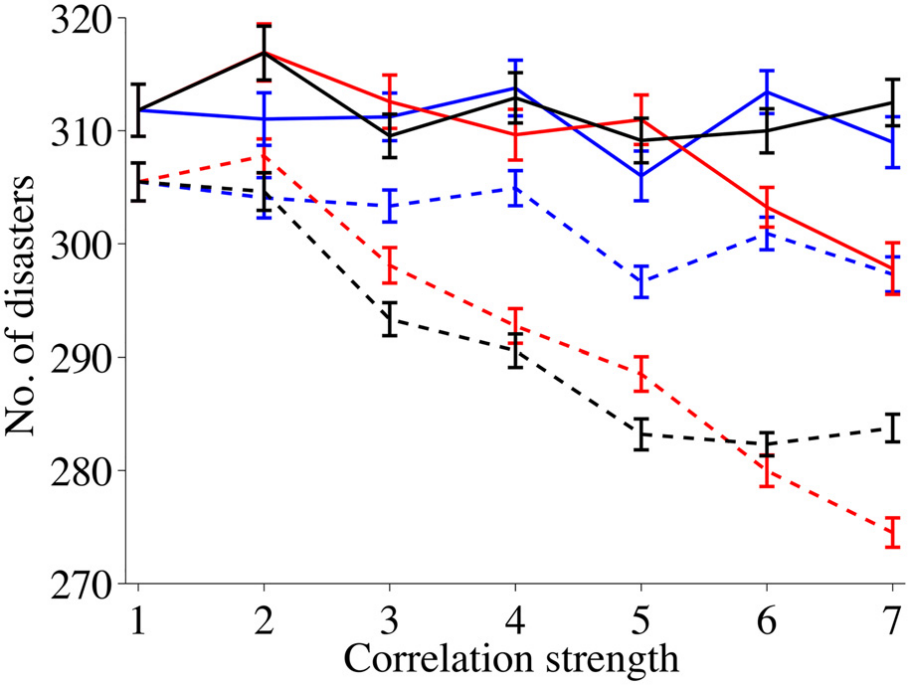
Number of disasters over a 50 year period that hit live nodes. Solid lines describe networks that are very inhomogeneous (*β* = 0.8), dashed lines describe networks that are more homogeneous (*β* = 0.2). Red curves describe spatial correlations among disasters, black curves represent spatial-temporal correlations and blue curves represent temporal correlations.

These qualitative arguments are supported by Fig 4 which shows the number of effective disasters, i.e. disasters that hit a live node as a function of *σ* and the various correlation cases. We see that homogeneous networks and spatially correlated disasters lead to a steady and dramatic reduction in effective disasters as the correlation strength increases. This is also true for inhomogeneous networks if the spatial correlation strength is high enough. In contrast, no masking effect can be discerned for inhomogeneous networks and temporal or spatio-temporal correlations whereas the impact of spatio-temporal correlation on homogeneous networks is much like the impact of spatial correlations.

**Fig 5:**
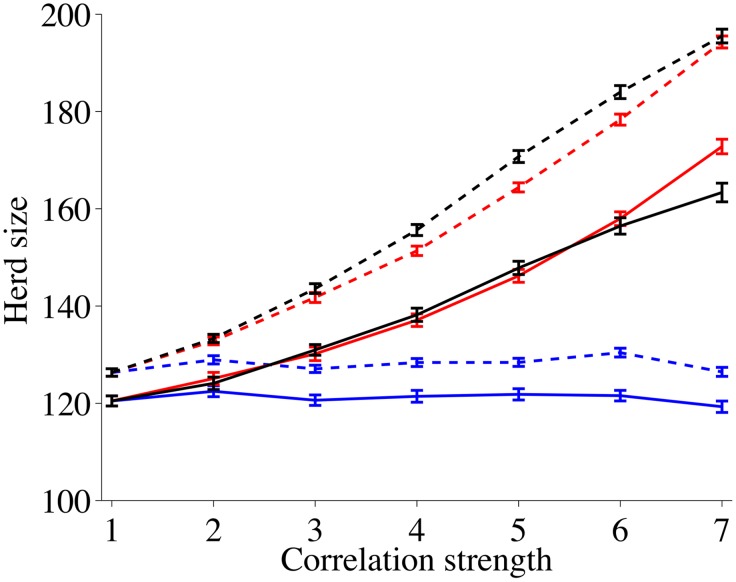
Average herd size per surviving node after 50 years as a function of the correlation strength among disasters. Solid lines describe networks that are very inhomogeneous (*β* = 0.8), dashed lines describe networks that are more homogeneous (*β* = 0.2). Red curves describe spatial correlations among disasters, black curves represent spatial-temporal correlations and blue curves represent temporal correlations.

*Masking* and its interaction with the homogeneity of the network has a profound influence on the wealth distribution of the surviving nodes, i.e. the average size of the herd after 50 years. Simulation using random and independent disaster events lead to 122 and 130 cattle per node for the highly inhomogeneous and the less inhomogeneous networks respectively. Those values stay the same for increasing temporal correlations. In contrast, increasing spatial and spatio-temporal correlations lead to a more than 50% increase of the average herd size to about 195 (165) cattle for inhomogeneous (homogeneous) networks (Fig 5). Notice that for a specific disaster correlation type, fewer survivors lead to larger surviving herds. However, this does not apply across correlations—temporally correlated disasters lead to the smallest number of survivors *and* to the smallest surviving herd sizes.

### Activated social structures

**Fig 6:**
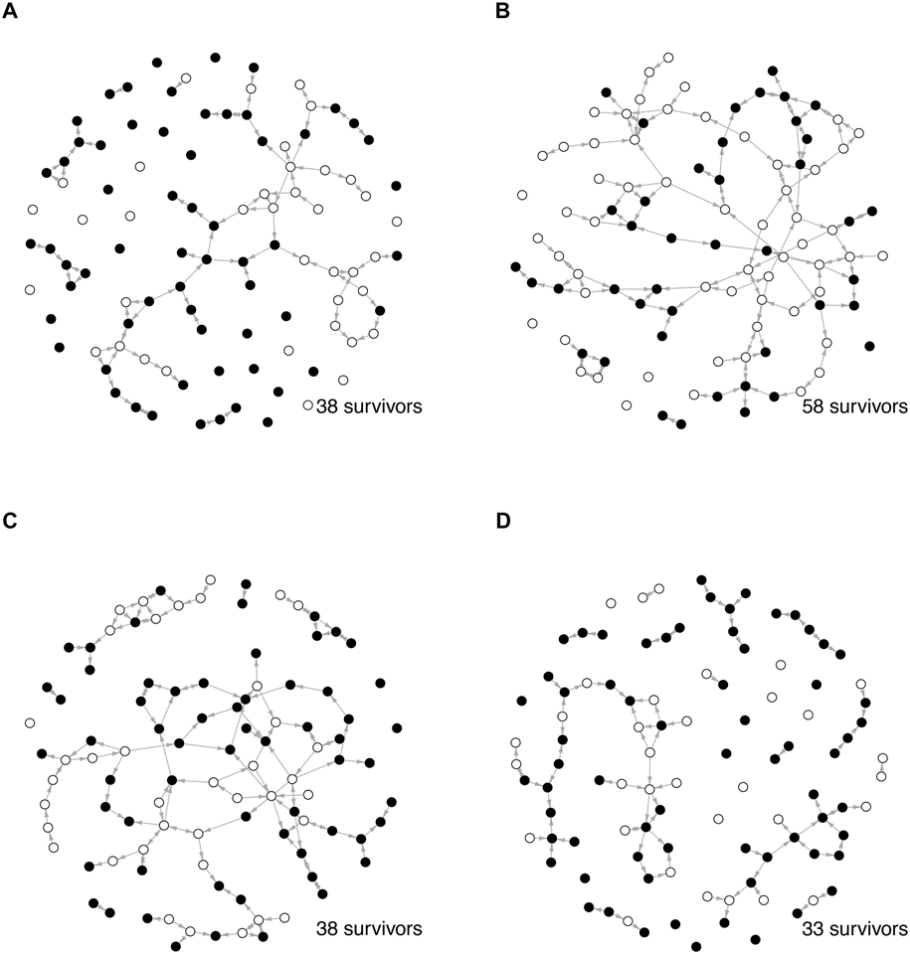
Four typical gift giving networks for *β* = 0.2 for a single simulation of 50 years. Red nodes are nodes that have survived, black nodes are dead. Directed links indicate one or more gift giving event in the course of a 50 year simulation.

We consider the Osotua network at the beginning of the simulation as the *potential* network, reflecting all the possible ways that support (cattle) may flow over the network. Registering all support events of a simulation gives us a cattle-flow network that represent the *activated* social network, i.e. the parts of the social structure represented by the *potential* network that was activated through the disasters. Fig 6 shows typical examples of these cattle flow networks for the four disaster correlation scenarios. Visual inspection shows that networks generated by disasters with spatio-temporal correlations typically lead to sparse networks where the few connected nodes form long open chain-like structures. Networks for the other cases are not easily distinguished visually from each other but clearly have higher connectivity. Another way of characterizing these flow networks is that the flow networks in the spatio-temporal correlation case are a disintegrated fraction of the potential network, whereas in the other cases, the flow network often represents the giant component of the potential network.

**Fig 7:**
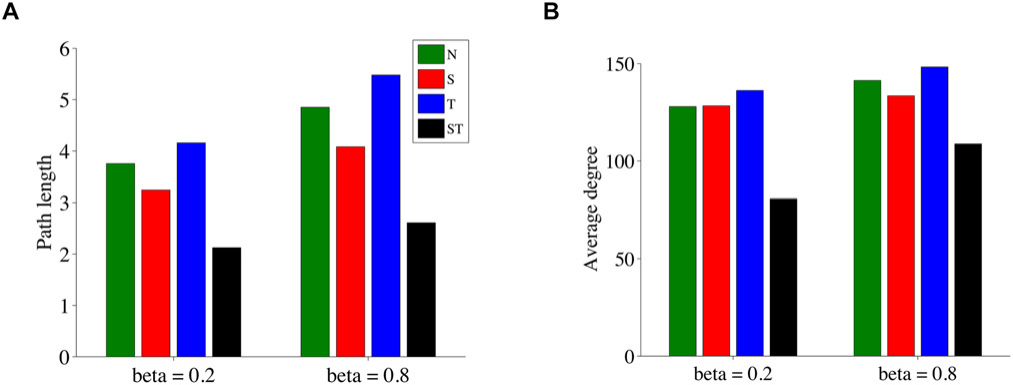
a) Average path length and b) average degree of the cattle flow networks for the four different disaster correlation cases.

To validate the visual impressions and to quantify the differences between the cases, we determine a set of graph properties for these cattle flow networks. Fig 7(a) and 7(b) show the average path length and the average degree for the four correlation schemes, both for almost homogeneous networks (*β* = 0.2) and for strong spatial network heterogeneity (*β* = 0.8). For both network types, the spatio-temporal scheme clearly stands out as particularly sparse, with a short path length and a low average degree. In contrast, the largest path length and the highest degree is shown by the networks generated from purely temporal correlations. Note that in calculating the average path length we ignored isolated nodes. Network clustering coefficients and the number of cycles in a network are presented in Table 1. Again, the spatio-temporal correlation case is very special: The number of cycles is much lower than in the other cases and for *β* = 0.2 the clustering coefficient is also much lower. For *β* = 0.8 the clustering coefficient for all three cases are the same. However, the number of cycles is markedly different for purely temporal correlations (57) vs. spatio-temporal correlations (9).

**Table 1:** Correlation, clustering and cycles.

correlation type	*β*	clustering coefficient	number of cycles
none	0.2	0.098 (0.018)	25 (59)
none	0.8	0.016 (0.023)	28 (584)
spatial	0.2	0.115 (0.018)	21 (31)
spatial	0.8	0.014 (0.021)	20 (25)
temporal	0.2	0.110 (0.019)	28 (146)
temporal	0.8	0.015 (0.246)	57 (6499)
spatio-temporal	0.2	0.044 (0.007)	4 (1.7)
spatio-temporal	0.8	0.012 (0.014)	9 (3.1)

Average clustering coefficient and average number or cycles for the different disaster scenarios and more (*β* = 0.2) or less (*β* = 0.8) homogeneous networks. The values in parentheses reflect the clustering coefficient and the number of cycles for randomized networks with the same number of nodes and links and the same degree distribution.

Comparing clustering coefficient and the number of cycles in the network for these flow networks with randomized networks with the same number of nodes and links and the same degree distribution we find in particular that the number of cycles in the network has dropped dramatically for the cattle flow network relative to the randomized network for purely temporal correlation whereas the same number has increased (albeit at very small numbers) for the spatio-temporal case.

**Fig 8:**
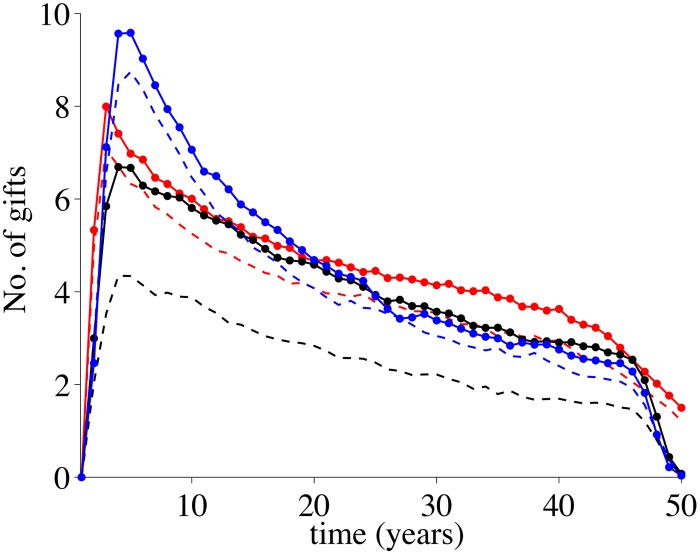
Gift giving events as a function of time. Solid lines describe networks that are very inhomogeneous (*β* = 0.8), dashed lines describe networks that are more homogeneous (*β* = 0.2). Red curves describe spatial correlations among disasters, black curves represent spatial-temporal correlations and blue curves represent temporal correlations.

Fig 8 illustrates the temporal sequence of gift giving events that lead to the flow networks in Fig 6 and to the results presented in Fig 7. We observe that in the spatio-temporal case for networks with low spatial heterogeneity (*β* = 0.2) very few gift giving events occur. For networks with higher spatial heterogeneity, however, the event numbers become comparable to those in the other scenarios, while the average degree increases only slightly which indicates a highly economical use of the gift giving network. In the temporal correlation case gift giving is concentrated at the beginning of the time interval whereas for the spatial case gift giving is concentrated at much later time.

## Conclusion

We have studied a highly stylized dynamical model of the impact of the strength and type of correlations of repeated disasters hitting a networked population. The defining features of the dynamical model are a small growth rate in the absence of disasters, limited network based resource transfers to mitigate disasters and thresholds to determine the viability of a population.

We find that for the same number of disasters, temporally correlated disasters are far more destructive than spatially correlated or spatio-temporally correlated disasters. In addition, temporal disasters effectively equalize the surviving populations at relatively low levels whereas spatial and spatio-temporal correlations lead to much larger surviving populations. We discuss the destructive capacity of disasters, ie. the number of disasters actually hitting a live node and show that a masking effect arising from an increasing number of disasters hitting empty sites is at least partially responsible for the higher survival rates in these cases. Spatial correlations enhance such a masking substantially whereas spatio-temporal correlations further contribute to this by providing rapid multiple hits at certain spatial sites (thus impeding the recovery of the site between subsequent disasters). It should be noted that the effect of masking strongly depends on interaction between the disaster dynamics, the viability threshold and the raw population growth rates (in particular the average number of disasters required to eliminate a site).

The architecture of the support network plays no role for temporally correlated disasters but has a strong impact on the destructive capacity of disasters otherwise. Having a very inhomogeneous support network enhances the ability to survive spatially and spatio-temporally correlated disasters by having a higher probability of unaffected nodes that can provide support.

In addition, the actual flow of support through the network creates its own network that can be considered a dual to the potential insurance network. We show that spatio-temporally correlated disasters lead to much more fractured support networks than all the other cases. To quantify this effect better and to understand its underlying mechanisms is the focus of future research.

Our study is related to the analysis of robustness of networks against errors and attacks e.g. [10]. However, in those studies, there is a functioning network, typically a communication network, which then may lose functionality (usually measured by a change in network diameter or by the fragmentation of the network) under a random failure of a node or through the targeted attack of a (e.g., hub) node. The important feature of the gift giving network, however, is its invisibility without disasters/attacks. Links are only evoked under the action of a disaster. Therefore, our study addresses the recovery of networks under attack, rather than their robustness. In addition, in contrast to a communication or a power system network, there is no central purpose to the network: Survival of a high percentage of the entire population may be a result of the gift giving insurance scheme but the measure of success of the insurance scheme is the probability of survival of the individual node.

Our results substantially agree with the theory of metapopulations [11] which studies the population dynamics of spatially separated populations in different ecological niches. In our case, spatial correlation of the disasters creates the ecological niches and the support network create immigration and emigration into different niches. We extend the metapopulation paradigm by extending the concept of ecological niches into the temporal domain and by replacing stochastic biological migration by a type of insurance scheme practiced by human groups. Our main conclusion translated into the ecological domain is that for the same number disasters, temporally correlated ones are more damaging to the survival of a species than just randomly and uncorrelated disasters and even much more damaging than spatially correlated ones and that both effects are reduced for highly mobile populations.

It is also instructive to speculate on the possible relationships of our results with the impact of global warming on human societies or ecological systems. As many natural disasters are highly correlated (tornado alley, draughts, landscapes vulnerable to flooding and mudslides, etc) there is an expectation of more and more correlated disasters as a result of global warming. Our results might suggest that disasters that are characterized predominantly by spatial structures like a rising sea-level are less destructive, due to either support transfers or due to abandoning of highly vulnerable regions, than temporally clustered events that are spatially more homogeneous like the global shift of climate zones. Disasters of the latter type may exhaust the possible resource transfers quickly and the affected communities do not have the option of escaping the disasters via relocation leading to a much higher overall impact than the former type.

## Future work

We have tried to study the impact of correlations on a dynamical system on a network in a generic and bare-bones model. However, we have just scratched the surface of this research theme. We present the fundamental modeling issues and discuss possible alternate choices to the ones we made that lead to future research:

- How is the network embedded into physical space? We chose to use a 1-d regular grid prior to rewiring to determine physical distance. Extensions to a 2-d regular grid with rewiring or to existing 2-d networks like the airline transportation network or the interstate highway network are clearly relevant for many social networks. A related question then is the choice of rewiring rules: should they be completely random as in our model or should the probability of a link between two distant nodes decay with the distance?
- What is the impact of a disaster? In our model disasters hit a node in a network and reduce a state variable associated with that node. For human infrastructure networks, it may be more appropriate to have disasters strike an edge or degrade the weight (e.g. the capacity for transport) of an edge.
- What is the size of the network? Increasing the number of the participants can be done in many ways: Since the number of participants is related to a spatial dimension via the initial grid size, we can choose to maintain the density in space and simultaneously increase space and node numbers. Assuming correlated disasters on such large scales also implies a choice for the type of disaster we are considering. Droughts or floods typically do not affect a whole continent. Alternatively, we could reduce the spatial distance with the increased number of nodes such that the spatial domain stays the same and the density increases. This would allow us to study the different impact of correlated disasters on low and high population densities.
- How do we compare the impact of spatial and temporal correlations? We have chosen to have the same number of disasters distributed in a spatially correlated and a temporally correlated way. When we increase the size of a network, the natural way from a temporally correlated perspective is to increase the number of disasters proportional to the increase of the number of nodes. Since we are dealing with transient dynamics, the perspective from a spatially correlated perspective is less clear: The transient dynamics introduces a time scale in which the disasters are relevant. Similarly to the interaction between network size and spatial scales, we can choose to keep the density of disasters constant by increasing their number *and* increasing the transient time scale, or we can choose to increase the temporal density of the disasters and study the interplay between the different time scales.
- What is the dynamics that is evolving on the network? Our dynamics is an exponentially growing population focussing on the initial growth phase. Other dynamical scenarios are a system in equilibrium or exponentially decaying populations. For instance, limiting the growth of the herds via a logistic growth model one could study the stability of the equilibrium population against different disaster scenarios.
- What is the functionality of the network? One unusual feature of our network is that it is an insurance scheme and that without disasters the network is invisible. Studying flow networks (like power systems, information exchanges, infections on social networks etc) that have very different functionalities will lead to very different ways of measuring the impact of disasters and hence may likely produce some very different results. Those networks are also very likely to be much larger than the networks we studied and hence the issues of scale free vs. random networks may become important as in [10].

## Supporting Information

S1 Text Supplement. We discuss how strongly our findings depend on network sizes (Supplement 1.1) and other topological properties like the degree distribution (Supplement 1.2), and the average degree spread and degree-degree correlations (Supplement 1.3).
(PDF)

S1 Fig Simulations 1000 nodes, small world networks. a) Average survival rates after 50 years b) number of disasters that hit live nodes over a 50 year period, c) average herd size per surviving node after 50 years as a function of the correlation strength of disasters. Simulations are performed with 1000 nodes. Solid lines describe networks that are very inhomogeneous (*β* = 0.8), dashed lines describe networks that are more homogeneous (*β* = 0.2). Red curves describe spatial correlations among disasters, black curves represent spatial-temporal correlations and blue curves represent temporal correlations.
(TIF)

S2 Fig Simulations 100 nodes, mean degree 4, power law networks. a) Average survival rates after 50 years b) number of disasters that hit live nodes over a 50 year period, c) average herd size per surviving node after 50 years as a function of the correlation strength of disasters. Simulations are performed with 100 nodes and a power law degree distribution with a mean degree of four. Red curves describe spatial correlations among disasters, black curves represent spatial-temporal correlations and blue curves represent temporal correlations.
(TIF)

S3 Fig Simulations 1000 nodes, mean degree 10, power law networks. a) Average survival rates after 50 years b) number of disasters that hit live nodes over a 50 year period, c) average herd size per surviving node after 50 years as a function of the correlation strength of disasters. Simulations are performed with 1000 nodes and a power law degree distribution with mean degree 10. Red curves describe spatial correlations among disasters, black curves represent spatial-temporal correlations and blue curves represent temporal correlations.
(TIF)

S4 Fig Scatter plots. Scatter plots for the average survival rate after 50 years for 1000 different network, (a) as a function of the maximal degree among all the nodes in the network, (b) as a function of the degree-degree correlation in the network. The former leads to a weak negative correlation, the latter to a weak positive correlation.
(TIF)
