# Supplementary material for: Node Survival in Networks under Correlated Attacks
Source: PLoS One. 2015 May 1;10(5):e0125467. doi: 10.1371/journal.pone.0125467 (PMC4416727; doi:10.1371/journal.pone.0125467)
Supplement: S1 Text — We discuss how strongly our findings depend on network sizes (Supplement 1.1) and other topological properties like the degree distribution (Supplement 1.2), and the average degree spread and degree-degree correlations (Supplement 1.3). (PDF) [file pone.0125467.s001.pdf]

Supporting Information Figure S1: Simulations 1000 nodes, small world networks

Figure 1: a) Average survival rates after 50 years b) number of disasters that hit live nodes over a 50 year period, c) average herd size per surviving node after 50 years as a function of the correlation strength of disasters. Simulations are performed with 1000 nodes. Solid lines describe networks that are very inhomogeneous ($\beta = 0.8$), dashed lines describe networks that are more homogeneous ($\beta = 0.2$). Red curves describe spatial correlations among disasters, black curves represent spatial-temporal correlations and blue curves represent temporal correlations.

1 Supplement

As a schematic model of the social systems investigated here, the setup presented in the main part of the text (N=100, $\langle k \rangle = 4$, ring graph with random link rewiring) is plausible. It represents a social structure of 100 herds, with an average number of four Osotua relationships each and the ring-like backbone graph representing a (one-dimensional) spatial embedding. In another paper [8] we have shown that there is a diminishing return to adding more nodes into an Osotua network.
Nevertheless, from a more theoretical perspective, it is an important question, how strongly our findings depend on network sizes (Supplement 1.1) and other topological properties like the degree distribution (Supplement 1.2), and the average degree spread and degree-degree correlations (Supplement 1.3).

1.1 Size dependence

We looked at larger networks which may be relevant in the context of other social or economic networks. We present the results of simulations with networks of 1000 nodes. Specifically, we keep the density of nodes in space the same and keep the probability to get hit by a disaster constant. Hence we increase the number of disasters by 10 and the groups of disasters also by 10. Previous research has shown [8] that the mean survival rate at 50 years is independent of the number of nodes for $N \geq\approx 50$. Therefore we did not change the temporal window. Hence the disasters are distributed in a

Supporting Information Figure S2: Simulations 100 nodes, mean degree 4, power law networks

Figure 2: a) Average survival rates after 50 years b) number of disasters that hit live nodes over a 50 year period, c) average herd size per surviving node after 50 years as a function of the correlation strength of disasters. Simulations are performed with 100 nodes and a power law degree distribution with a mean degree of four. Red curves describe spatial correlations among disasters, black curves represent spatial-temporal correlations and blue curves represent temporal correlations.

Supporting Information Figure S3: Simulations 1000 nodes, mean degree 10, power law networks

Figure 3: a) Average survival rates after 50 years b) number of disasters that hit live nodes over a 50 year period, c) average herd size per surviving node after 50 years as a function of the correlation strength of disasters. Simulations are performed with 1000 nodes and a power law degree distribution with mean degree 10. Red curves describe spatial correlations among disasters, black curves represent spatial-temporal correlations and blue curves represent temporal correlations.

rectangle of size 1000×50. The resulting curves showing mean herd survival rates after 50 years, (Supp. Fig. 1a), the number of effective disasters (Supp. Fig. 1b), and surviving herd size (Supp. Fig. 1c) for spatially, temporally and spatio-temporally correlated disasters are smoother than the curves in Figures 3-5 but are qualitatively the same.

1.2 Power law distributions

While nothing is known about the topological type of network for the Osotua network, typical other social networks can often be represented by power law distributions. Specifically disease transmission networks seem to have power law distributions and may be a good proxy for the insurance networks [12,13]. To determine the influence of the degree distributions of the Osotua networks on the survival rates we generate random networks with a power law distribution via preferential attachment fixing the mean degree. We keep our spatial arrangement on a ring, giving every node a

spatial location. Supp. Fig. 2 shows mean survival rate at 50 years, number of effective disasters and surviving herd size for the three cases of disaster correlations for networks of 100 nodes with mean degree four and a power law distribution. Supp. Fig. 3 shows the same curves for networks of 1000 nodes and a mean degree of 10. The generation of the 1000 disasters follows the discussion in Supplement 1.1. We find that, as noted already in [8], all survival rates improve if the mean degree increases. In addition, comparing the curves for effective disasters for power law distribution networks with the spatially homogeneous and inhomogeneous small world networks, we find that a power law distribution with high mean degree generates spatial inhomogeneity such that such a network behaves similarly to the small world network with high spatial heterogeneity ($\beta = 0.8$). Beyond that finding, the impact of the correlations of the various types of disasters on the survival rates and the wealth distribution of the survivors are qualitatively the same for the small world networks discussed in section 4 and the power law networks, confirming the robustness of our main conclusions.

1.3 Other topological features

From the multitude of additional topological features that could in principle affect the survival rate, we looked at the width of the degree distribution and degree-degree correlations. We took the data from the two simulations: i) the small world graph of 100 nodes and mean degree of four and ii) the power law graph of 1000 nodes and mean degree of ten. We determine the correlation between the maximal degree among all the nodes for a given sample of a small word network or a power law network, using it as a surrogate for the width of the degree distribution and the corresponding network survival rate.

For random disasters without correlation and for a rewiring coefficient $\beta = 0.8$, Supp. Figure 4(a) shows a scatter plot for 1000 different networks with the average survival rate and the maximal degree of a node in the network as the two axes. The figure shows a weak negative correlation between survival rate and maximal degree. Supp. Table 1 shows the same weak correlation for other disaster types. In addition, the same table shows that the anti-correlation between the largest degree and survival becomes a bit more pronounced for the power law distributions, suggesting that highly centralized networks are correlated with lower survival rates.

Regarding their assortativity (i.e., their amount of degree-degree corre-

Supporting Information Table S1: Correlations

correlation type	survival rate vs. max degree	survival rate vs. assortativity.
none	-0.25/ - 0.388	0.18/0.513
spatial	-0.226/-0.415	0.177/0.542
temporal	-0.232/-0.377	0.172/0.540
spatio-temporal	-0.223/-0.375	0.192/0.505

Table 1: Correlations coefficients for the survival rate with the max degree and the assortativity. The first number for each table entry is for the simulations represented in the scatter plots in Supp. Figure 4 for the four different disaster correlation scenarios . The second number is for the same analysis for the power law distributions discussed in Supplement 1.2.

lations), some of these networks display slightly positive degree correlations, while others show slightly negative correlations. Furthermore, for a given disaster scenario, each network is characterized by its average survival rate. We can thus compute the correlation coefficient between the networks assortativity and its average survival rate: Supp. Figure 4(b) shows a scatter plot

Supporting Information Figure S4: Scatter plots

Figure 4: Scatter plots for the average survival rate after 50 years for 1000 different network, (a) as a function of the maximal degree among all the nodes in the network, (b) as a function of the degree-degree correlation in the network. The former leads to a weak negative correlation, the latter to a weak positive correlation.

for survival rate vs. degree-degree distribution (assortativity). The correlation is weakly positive. Supp. Table 1 shows the same weak correlation for the small world networks and a stronger positive correlation for the power law networks for all disaster schemes, respectively. The implications of these findings and a causal narrative for them are interesting questions that are however, not the focus of the current study.
