# Supplementary figures and images for: Node Survival in Networks under Correlated Attacks
Source: PLoS One. 2015 May 1;10(5):e0125467. doi: 10.1371/journal.pone.0125467 (PMC4416727; doi:10.1371/journal.pone.0125467)

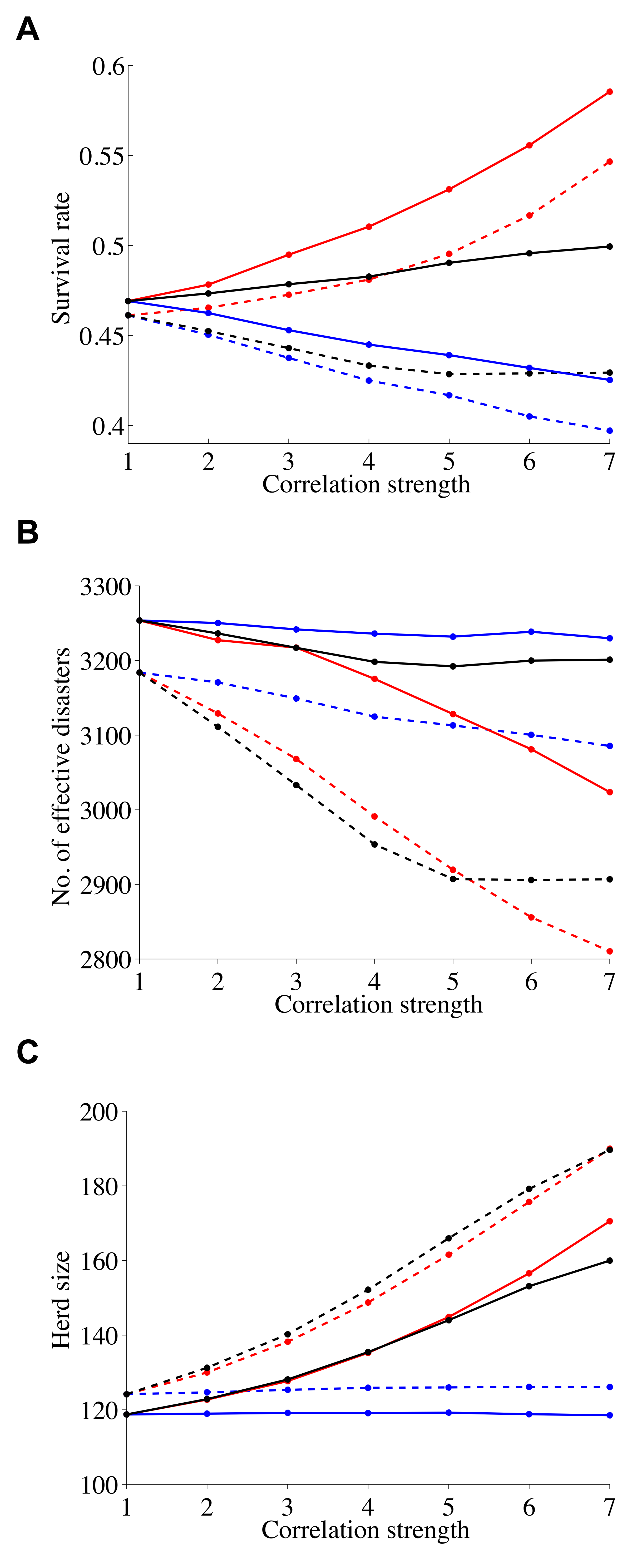

Supplement: S1 Fig — a) Average survival rates after 50 years b) number of disasters that hit live nodes over a 50 year period, c) average herd size per surviving node after 50 years as a function of the correlation strength of disasters. Simulations are performed with 1000 nodes. Solid lines describe networks that are very inhomogeneous (β = 0.8), dashed lines describe networks that are more homogeneous (β = 0.2). Red curves describe spatial correlations among disasters, black curves represent spatial-temporal correlations and blue curves represent temporal correlations. (TIF) [file pone.0125467.s002.tif]

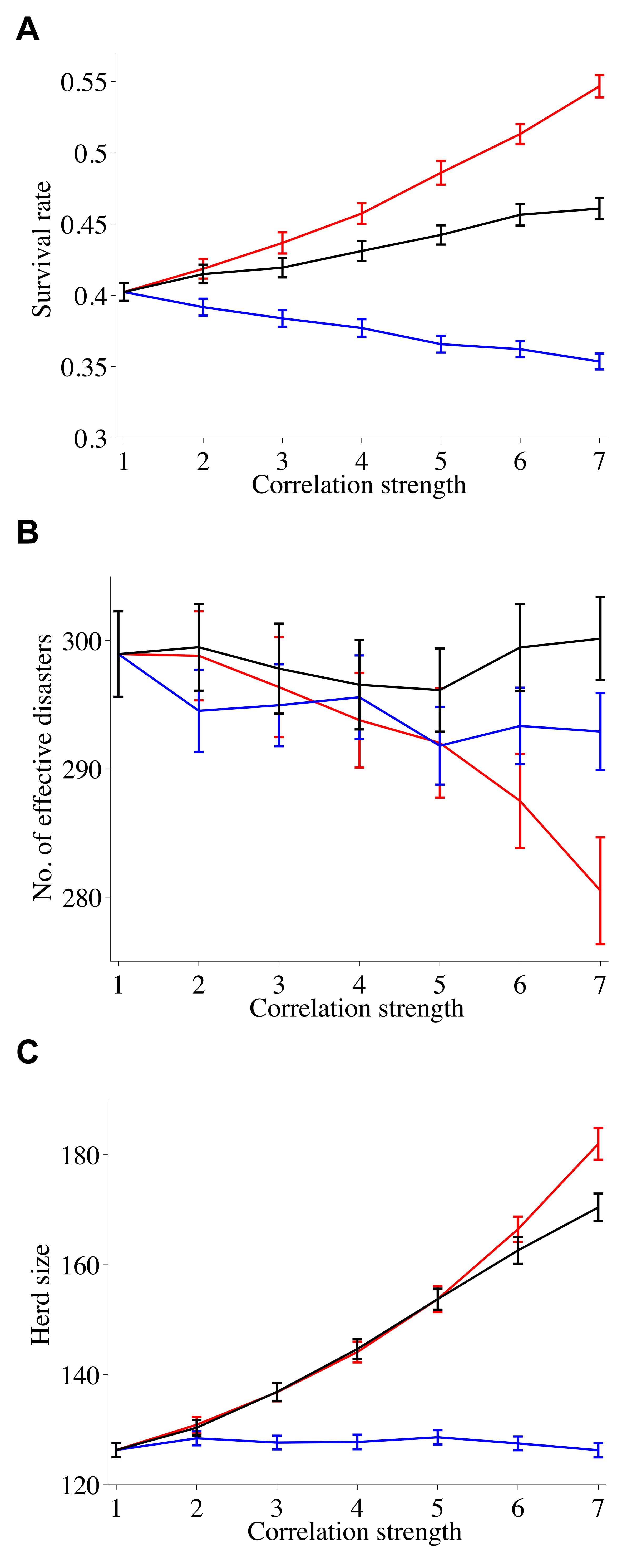

Supplement: S2 Fig — a) Average survival rates after 50 years b) number of disasters that hit live nodes over a 50 year period, c) average herd size per surviving node after 50 years as a function of the correlation strength of disasters. Simulations are performed with 100 nodes and a power law degree distribution with a mean degree of four. Red curves describe spatial correlations among disasters, black curves represent spatial-temporal correlations and blue curves represent temporal correlations. (TIF) [file pone.0125467.s003.tif]

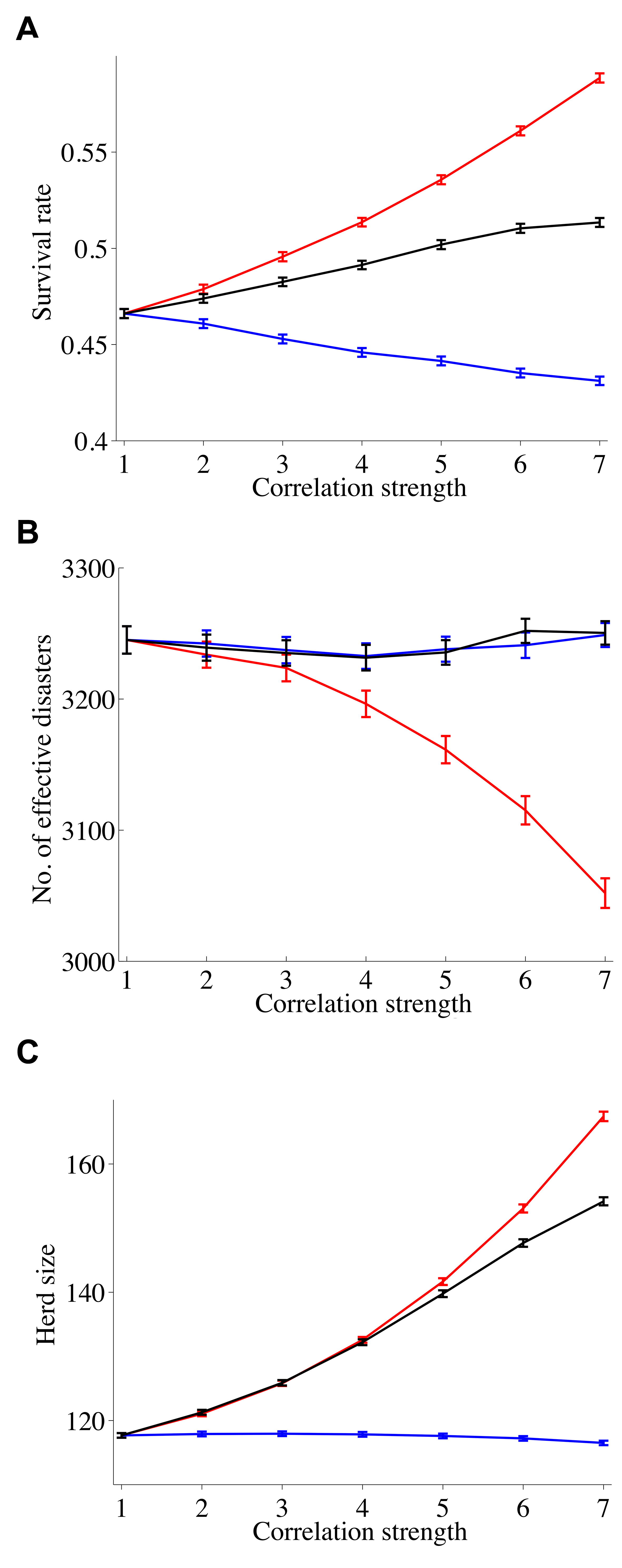

Supplement: S3 Fig — a) Average survival rates after 50 years b) number of disasters that hit live nodes over a 50 year period, c) average herd size per surviving node after 50 years as a function of the correlation strength of disasters. Simulations are performed with 1000 nodes and a power law degree distribution with mean degree 10. Red curves describe spatial correlations among disasters, black curves represent spatial-temporal correlations and blue curves represent temporal correlations. (TIF) [file pone.0125467.s004.tif]

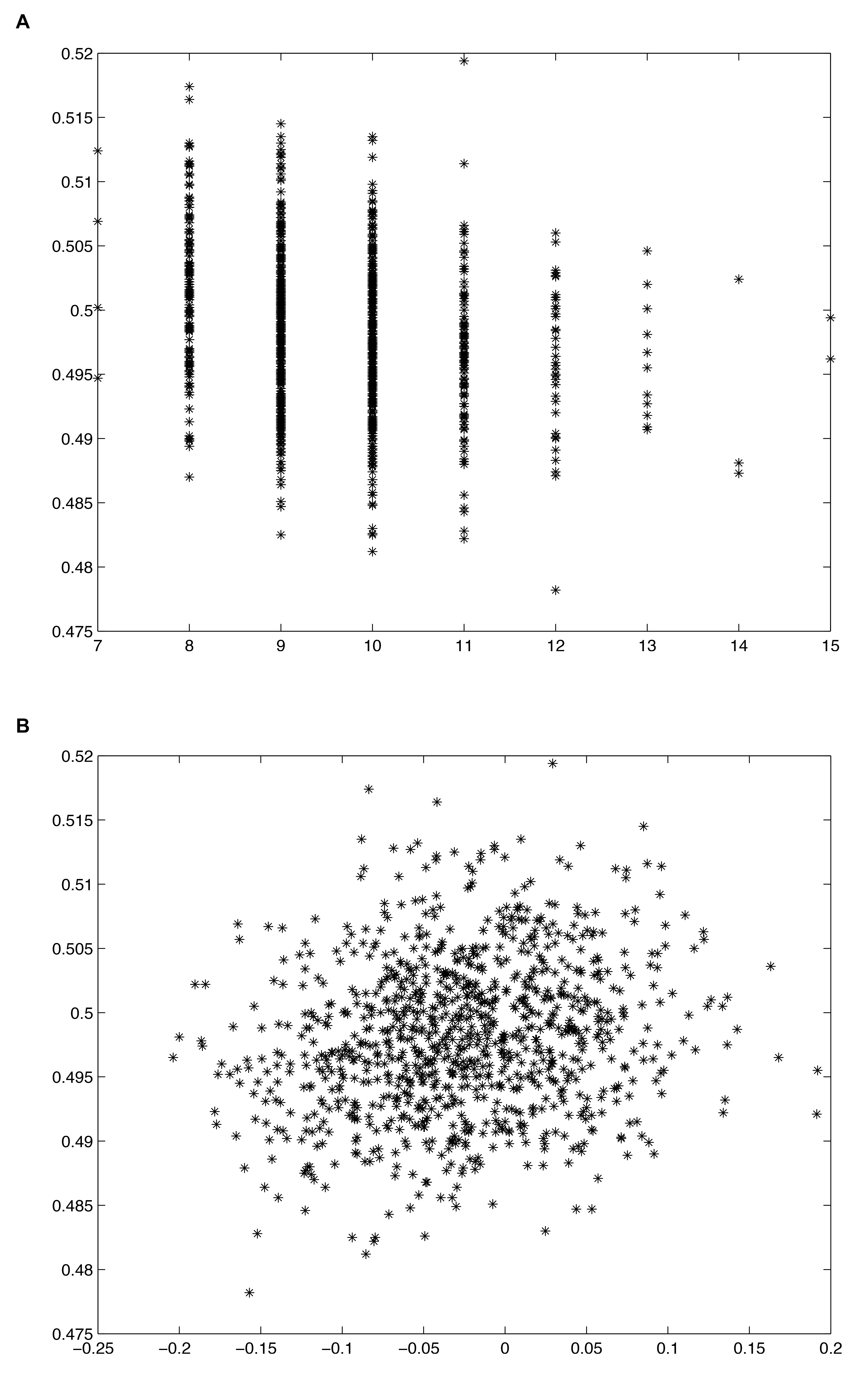

Supplement: S4 Fig — Scatter plots for the average survival rate after 50 years for 1000 different network, (a) as a function of the maximal degree among all the nodes in the network, (b) as a function of the degree-degree correlation in the network. The former leads to a weak negative correlation, the latter to a weak positive correlation. (TIF) [file pone.0125467.s005.tif]
